# A Review on Polymer Precursors of Carbon Molecular Sieve Membranes for Olefin/Paraffin Separation

**DOI:** 10.3390/membranes11070482

**Published:** 2021-06-29

**Authors:** Seong-Joong Kim, YongSung Kwon, DaeHun Kim, Hosik Park, Young Hoon Cho, Seung-Eun Nam, You-In Park

**Affiliations:** 1 Green Carbon Research Center, Korea Research Institute of Chemical Technology (KRICT), Gajeong-ro 141, Yuseong-gu, Daejeon 34114, Korea; joong@krict.re.kr (S.-J.K.); yskown@krict.re.kr (Y.K.); daehun72@krict.re.kr (D.K.); hspark@krict.re.kr (H.P.); yhcho@krict.re.kr (Y.H.C.); senam@krict.re.kr (S.-E.N.); 2 Department of Chemical and Biomolecular Engineering, Korea Advanced Institute of Science and Technology (KAIST), Daejeon 34141, Korea; 3 Department of Chemical and Biological Engineering, Korea University, 5-1 Anam-dong, Seongbuk-gu, Seoul 02841, Korea

**Keywords:** olefin/paraffin, carbon molecular sieve membrane, polymer, precursor, pyrolysis

## Abstract

Carbon molecular sieve (CMS) membranes have been developed to replace or support energy-intensive cryogenic distillation for olefin/paraffin separation. Olefin and paraffin have similar molecular properties, but can be separated effectively by a CMS membrane with a rigid, slit-like pore structure. A variety of polymer precursors can give rise to different outcomes in terms of the structure and performance of CMS membranes. Herein, for olefin/paraffin separation, the CMS membranes derived from a number of polymer precursors (such as polyimides, phenolic resin, and polymers of intrinsic microporosity, PIM) are introduced, and olefin/paraffin separation properties of those membranes are summarized. The effects from incorporation of inorganic materials into polymer precursors and from a pyrolysis process on the properties of CMS membranes are also reviewed. Finally, the prospects and future directions of CMS membranes for olefin/paraffin separation and aging issues are discussed.

## 1. Introduction

Light olefins such as C_2_H_4_ and C_3_H_6_ are extremely important products in the petrochemical industry due to their uses in a variety of materials that include such as polyethylene, polypropylene, ethylene glycol, and isopropanol [1,2,3]. In particular, the polyolefins are major raw materials used in the production of plastics, fabrics, rubber, paints, cosmetics, and adhesives [4,5,6]. Generally, light olefins are manufactured using a naphtha cracking process, coal/methanol to olefin (CTO/MTO), or a propane dehydrogenation (PDH) process [7,8,9,10]. To produce plastics and polymers from olefins, their purity should be >99.9% [11,12]. However, light olefins are commonly accompanied with paraffin, and an additional process is needed to separate them [13,14].

To separate olefins from paraffin, which have similar molecular properties (e.g., boiling point (bp): C_2_H_6_ (−88.5 °C), C_2_H_4_ (−102.4 °C), C_3_H_8_ (−42.2 °C), and C_3_H_6_ (−47.7 °C)), energy-intensive cryogenic distillation at high pressure and low temperature with 150–200 trays has generally been employed [15,16]. Furthermore, these systems must use large compressors and heat exchangers that are expensive to build and to operate, while only being beneficial for producing olefin of high purity [17]. According to the literature [18], the energy consumed to separate olefins from paraffin (both C_2_H_4_ and C_3_H_6_) accounts for up to $5 billion USD per year. Moreover, the large space needed for such a system, as well as its high cost, is required due to its tall columns (>300 ft) and large number of trays.

Such cryogenic distillation, with its enormous cost and space, should be replaced by effective alternative energy and sustainable technologies. Extractive distillation is a common process for olefin/paraffin separation, but it is not better than traditional distillation [19]. For the past several decades, adsorption processes have been investigated for use in olefin/paraffin separation [20]. An equilibrium adsorption step followed by thermal regeneration variable-temperature stepwise desorption (VTSD) could be selective for C_3_H_6_ over C_3_H_8_ [21]. However, its capital cost is higher than the traditional distillation process while the energy cost of VTSD was lower. Recently, cyclic adsorption processes such as simulated moving bed (SMB), vacuum pressure swing adsorption (VPSA), and thermal swing adsorption (TSA) have been proposed. The successful performance of these technologies is intrinsically dependent on the selection of particular adsorbents such as silica-gel, 13X zeolite, 4A zeolite, 5A zeolite, and carbon molecular sieve (CMS) [22,23,24].

**Figure 1:**
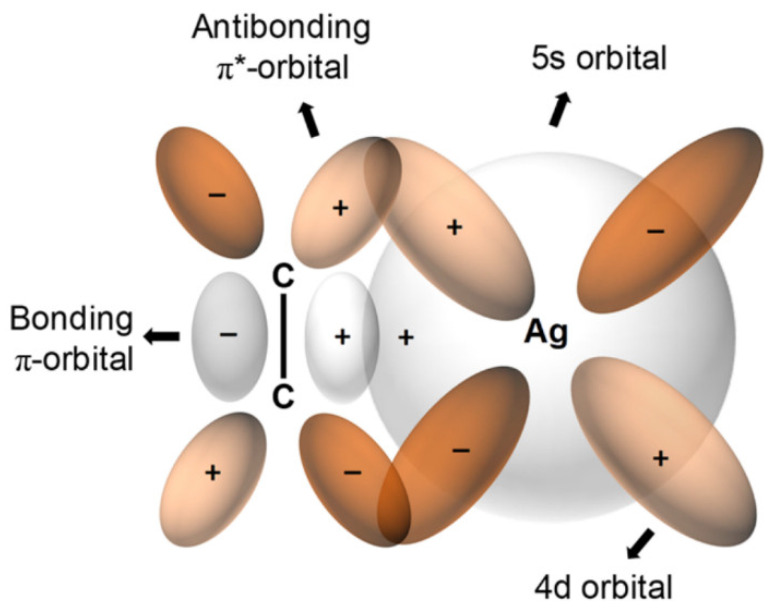
Coordination between a transition metal and olefin. Adapted from Ref. [25] with permission. Copyright (2018) American Chemical Society.

For olefin facilitated transport, transition metal cations are commonly used as carriers, thereby providing the ability to form reversible complexes of molecules with double bonds. Even though any transition metal could be used as a complexing agent, Ag^+^ is the most frequently employed due to the lower stability of Ag^+^-olefin complexes (relative to those of other metal-olefin transition complexes). This relative instability allows olefin to be released more easily. Ag^+^-olefin complexes include the following: an overlap between the occupied π-orbital of the olefin and the empty 5s-orbital of the Ag^+^ and between a π-bond offered by the back-donation of electrons from the occupied d-orbitals of the silver and the empty antibonding π*-orbitals of the olefin, as indicated in Figure 1 [19].

For olefin/paraffin separation, these separation technologies have great potential as alternatives to traditional distillation processes in terms of lower capital, operating, and energy costs. However, only a few non-distillation processes are being utilized in the petrochemical industry because these alternative processes still have inherent problems [26].

Membrane technologies offer attractive alternatives that could reduce the large capital and high operating costs of the cryogenic distillation process for olefin/paraffin separation. Sholl and Lively stated that membrane-based separation would use 90% less energy than distillation [27]. Therefore, a number of articles have been published on olefin/paraffin separation with membrane technologies [28,29,30,31,32,33]. Many researchers are investigating this notion using available membranes and processes to enhance the performance of olefin/paraffin separation in terms of possible commercialization. The membrane technologies for olefin/paraffin separation can be classified into three categories: facilitated-transport, polymeric, and inorganic membranes.

**Figure 2:**
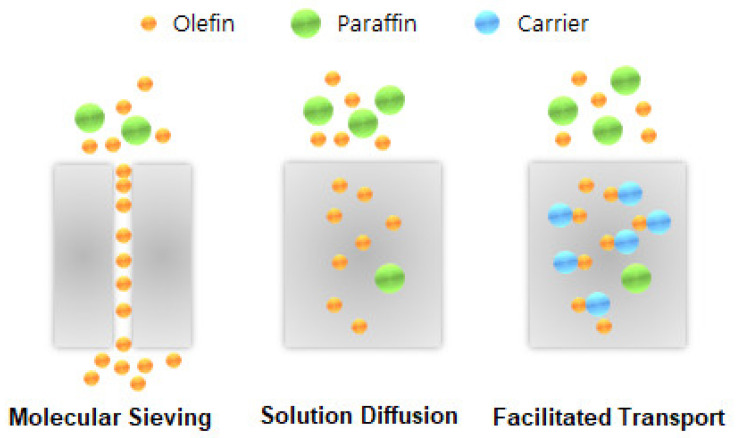
Molecular sieving, solution diffusion, and facilitated transport mechanisms.

The facilitated-transport membrane is one of the most favorable and important membranes for olefin/paraffin separation [33,34,35,36]. As shown in Figure 1, metal ions are able to form reversible chemical bonds with olefins due to the π-bonding between the hybrid molecular orbitals of olefin and the metal atomic orbitals. As shown in Figure 2, this chemical complexation between transition metal ions and olefins offers high selectivity and high capacity. This is due to bonding that is relatively stronger than Van der Waals forces, but still weak enough to be broken by moderate increases in the temperature or decreases in the pressure [37]. Studies have also been done utilizing membranes with a variety of solvents for olefin absorption. The facilitated transport membranes can be operated as liquid membranes and membrane electrolytes for liquid and solid states, respectively. However, the inherent problems of facilitated transport membranes, such as short life span due to carrier poisoning, should be addressed for better commercial application [38,39].

Polymeric membranes including a variety of materials have been extensively investigated for olefin/paraffin separation [29,30,40]. The separation performance of polymeric membranes is mostly determined by polymer properties such as molecular weight, shape, polymer structure, packing, and rigidity. Among the various polymers, glassy polymers show better performance for olefin/paraffin separation, as well as for the separation of aromatic, alicyclic, and aliphatic hydrocarbons. Rubbery polymers are appropriate for hydrocarbon/air separation and pervaporation processes such as hydrocarbon separation from aqueous solutions [41]. Large-scale polymeric membranes are widely used in the markets of the world for separation processes due to the availability of low-cost polymer materials. However, it is very difficult to separate olefin from paraffin using a polymeric membrane due to the limitation of trade-off between permeability and selectivity and parasitic plasticization effects. Furthermore, polymeric membranes are not suitable for application in harsh environments [42,43].

**Figure 3:**
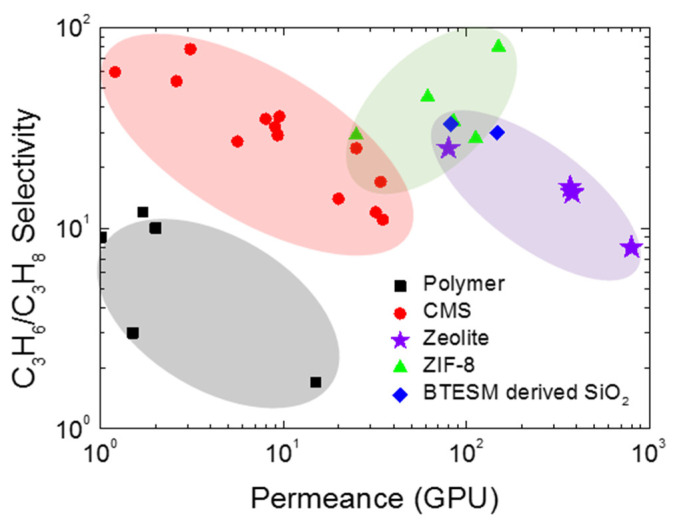
Comparison of polymeric membranes and inorganic membranes for C_3_H_6_/C_3_H_8_ separation.

The inorganic membranes have received a great deal of attention as replacements for polymeric membranes. To surpass the upper bound trade-off for olefin/paraffin separation, recent researchers have been focusing on the development of inorganic membranes, in particular, microporous membranes such as zeolite, silica, zeolitic imidazolate frameworks (ZIFs), and CMS membranes (Figure 3) [25,44,45,46,47].

A zeolite membrane has an effective layer with a uniform pore structure of molecular sieving units, relative to those of other inorganic materials [48]. However, the status of research on zeolite membranes for olefin/paraffin separation is still laboratory scale. This is because synthesizing thin-film zeolite membranes is very difficult to do without defects, more of which tend to form as the membrane surface area increases [49]. Moreover, zeolite membranes are much more expensive than polymeric membranes and have poor mechanical stability [50,51].

ZIFs consisting of transition-metal ions interconnected by imidazolate linkers similar to the Si–O bond in zeolites, exhibit thermal and chemical stability. The pore size of ZIFs is distributed in the range 3–5 Å, providing molecular sieving separation appropriate for gas molecules. Two types of ZIF membranes have been prepared: mixed matrix membranes (MMMs) made by incorporating ZIF particles in a polymer matrix, and continuous thin-film membranes on porous supports. In particular, the continuous ZIFs membranes can offer the full intrinsic potential of ZIFs and achieve excellent separation performance. These membranes have recently been investigated by many researchers who focused on thin, defect-free supported membranes [52,53,54].

Amorphous silica has sufficiently small pore structure (3–3.5 Å) to serve as a kinetically selective material. The silica membranes are prepared via chemical vapor deposition (CVD) and a sol-gel method. They are applied for H_2_/He separation at high temperature, and show dramatically improved permselectivity [55,56,57]. However, silica membranes have the problem of an unstable amorphous structure when exposed to water vapor, leading to degradation of separation due to enlargement of their dense and amorphous structure [58].

On the other hand, CMS membranes for olefin/paraffin separation have favorable performance compared with ordinary polymeric membranes [59,60]. The CMS membranes are typically prepared using pyrolysis of polymeric precursors at high temperature under O_2_-free conditions. After carbonization of the polymer precursors, the structure of CMS membranes is significantly changed to form a rigid pore wall, which gives high gas permeation and selectivity with better thermal and chemical stability [61,62]. For commercialization and practical application of CMS membranes in industrial fields, some efforts have been attempted. Air products in the early 1990s developed the nanoporous carbon membranes called a selective surface flow membrane (SSFTM). The membrane consisted of thin layer of 2–3 μm onto alumina tubular support, suiting for the hydrogen/hydrocarbon separation. However, this was discontinued in 2003 due to the aging and deactivation of membranes in the presence of water vapor [63]. Carbon Membranes Ltd. (Beer-Sheva, Israel) also produced large-scale CMS membrane module (fibers of 10,000 strands and 4 m^2^/module with the packing density of 2000 m^2^/m^3^) by carbon CVD using propylene as the source into the bore side of fiber bundles after pyrolysis. It was but closed in 2001 [64]. Blue Membranes GmbH (Saerbeck, Germany) developed a new CMS membrane module, which was a flat type membrane of a honeycomb configuration. The patterned sheets were overlapped and sealed to create cross flow channels of the module. The packing density of the membrane modules was 2500 m^2^/m^3^ in a 10 m^3^ module [65]. Media and Process Technology Inc. (Pittsburgh, PA, USA) developed an 86-tube CMS membrane bundle/housing for hydrogen separation and contaminants removal from coal- and biomass-derived raw syngas. The membrane bundle was tested from the laboratory to the field-level. During field-testing over several hundred hours, the stable membrane performance was exceptionally observed without pre-treatment [66]. CMS membrane module with the surface area of 0.5–2.0 m^2^ was applied for the production of vehicle fuel from biogas. The membrane module achieved 97 mol% CH_4_ purity and 98% CH_4_ recovery [67]. However, comprehensive work on CMS membranes for olefin/paraffin separation is still inadequate and more is required.

## 2. Structure and Gas Transport Mechanism of a CMS Membrane for Olefin/Paraffin Separation

A CMS membrane was produced by the pioneers Barrer and Strachan in early 1955. A microporous plug was prepared by compressing a high specific-surface area carbon powder to study the adsorption and diffusion of gases. From this, it was confirmed that surface flow is important for most polarizable species [68,69]. Barrer and coworkers then comprehensively investigated the gas and vapor sorption and diffusion properties [70] of the compacted carbon membranes, which showed excellent intrinsic performance for gas separation. Most CMS membranes are currently achieved using a process involving pyrolysis of polymeric precursors at high temperature in an O_2_-free condition. It is considered a high free-volume material [71].

In particular, CMS membranes have attracted considerable attention for olefin/paraffin separation [62,72]. The rigid pores of CMS membranes enable penetration of gas molecules with impressive permselectivity and without deformation and deterioration of the pore structure during the hydrocarbon separation. Moreover, olefin/paraffin separation often requires harsh operating conditions (polymeric membranes show abnormal behavior in this specific situation [73,74]). CMS membranes are also cost-effective and easier to process than zeolite, metal-organic frameworks (MOFs), and silica membranes [75].

CMS membranes consists of a rigid and amorphous structure with small pore size distribution [76,77]. The pore structure of a CMS membrane has ultramicropores (0.3–0.5 nm), which allow molecular-sieving separation. However, larger micropores (0.6–2 nm) are also present and permit excellent penetration of gas due to their high sorption coefficient, which provides both high permeability and selectivity [78,79]. To allow gas diffusion by the molecular sieve mechanism, the molecules adsorbed in micropores must have more activation energy that is required to overcome repulsion from the walls of ultramicropores [64,80].

To examine the micropore structure and pore size of CMS membrane, several characterization technologies have been employed. The XRD patterns can provide the d-spacing value between basal planes of CMS membrane. This distance can be a transport pathway for the gas molecules, which help measure the change of carbon structure at different preparation process or polymer precursors. However, this is not indicative of pore size. CO_2_ or N_2_ physisorption is the most frequently used method to characterize the pore size of CMS membrane. In particular, CO_2_ physisorption using the density functional theory (DFT) can qualitatively describe the ultramicropore size distribution of CMS membrane. Besides, the pore size distribution of CMS membrane obtained from positron annihilation lifetime spectroscopy (PALS) has been often reported [81,82,83]. Positrons can either annihilate or trap in pores of CMS membrane. Therefore, the average positron lifetime increases with increasing pore size. In general, the results obtained from PALS is consistent with that of XRD. To measure the ultramicropores of CMS membrane, an approach based on gases of different size as molecular scale probes was designed. This provides the structure-performance relationships of CMS membrane [81].

**Figure 4:**
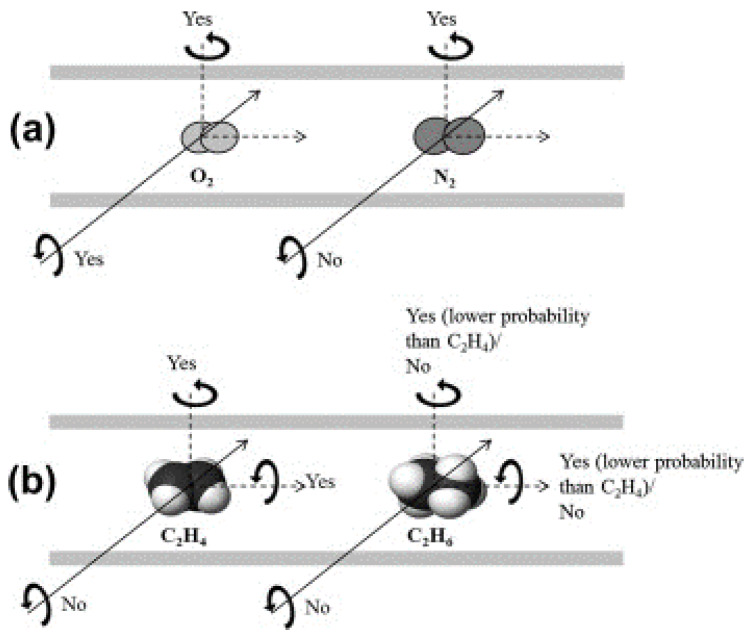
Illustration of the rotational degrees of freedom in the activated state through a slit-shaped pore of CMS: (**a**) O_2_/N_2_ system and (**b**) C_2_H_4_/C_2_H_6_ system. Adapted from [60] with permission. Copyright (2012) Elsevier.

On the other hand, Rungta et al. explained well the distinguishing features between CMS and zeolite membranes in terms of molecular sieve [84]. The CMS membrane consists of a slit-like pore structure with a one-dimensional (1-D) size restriction while the zeolite has open pores with 2-D size restriction. This unique pore structure of CMS membranes enables olefin, with a rather more planar molecular configuration than paraffin, to penetrate easily and effectively through the slit-like pores. In contrast, the specific configurational property of an olefin is meaningless to the open pores of zeolite. From this perspective, CMS membranes for olefin/paraffin separation are more theoretically ideal than zeolite is. The researchers also described the difference in entropic selectivity between O_2_/N_2_ and C_2_H_4_/C_2_H_6_ [60]. Both O_2_ and N_2_ can pass through the slit-like pore structure in the diffusion direction. However, the planar C_2_H_4_ molecule has higher probability of passing through the CMS pore while C_2_H_6_ may either need greater effort to pass through or may get rejected, as shown in Figure 4.

## 3. Polymer Precursors for CMS Membranes

**Figure 5:**
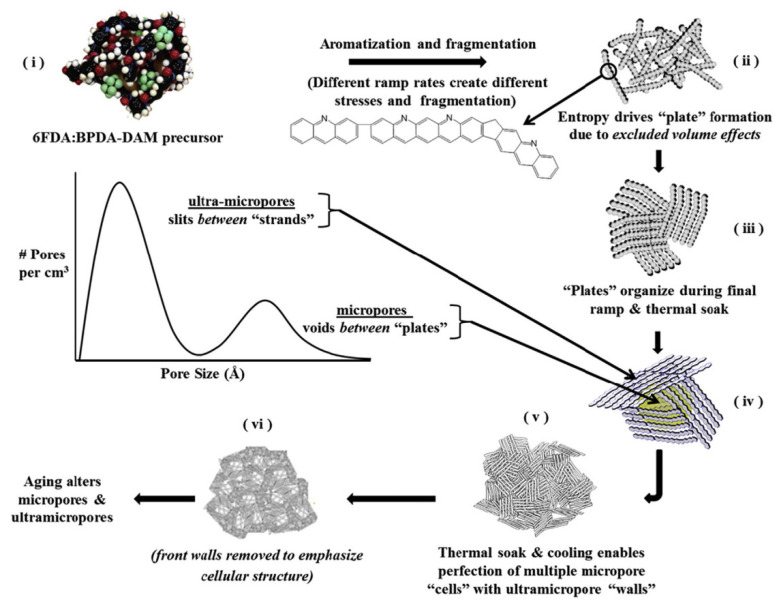
Illustration of the transformation steps of polyimide precursor to amorphous CMS material with micropores and ultramicropores. Adapted from Ref. [85] with permission. Copyright (2017) Elsevier.

A variety of polymer precursors can produce different outcomes in terms of the structure and performance of a CMS membrane. However, it is expected that the transition process from polymer chain to amorphous or turbostratic carbon structure during pyrolysis may be similar among most of the polymer precursors because they undergo the same heat-derived processes (such as ramping, soaking, and cooling) in sequence [61,80]. There is a progression from amorphous chains of the polymer precursor to the disordered stacked plates of carbon structures during the pyrolysis process. Rungta et al. [85] systematically and logically rearranged a pyrolysis protocol with 4,4′-(hexafluoroisopropylidene) diphthalic anhydride/3,3′-4,4′-biphenyl tetracarboxylic dianhydride-2,4,6-trimethyl-1,3-phenylene diamine (6FDA/BPDA-DAM) as polymer precursor, as indicated in Figure 5.

To obtain a stable and desirable CMS membrane in terms of separation performance and structure, the polymer precursor is an important parameter. The preferred precursors for CMS membranes typically exhibit heat-resistance property without considerable deformation during the required pyrolysis. During pyrolysis, the polymer precursor goes through a structural rearrangement process as follows: thermal decomposition and deformation, condensation, and aromatization, even though the polymer has a thermosetting property [86]. Nevertheless, the thermoplastic and lower molecular weight polymers have higher chain mobility and fractional free volume. Adjusting the molecular weight of the polymer precursors is a simple method by which to affect the degree of structural rearrangement and pore formation, and to determine the separation properties of the CMS membrane [86,87]. Herein, we review several representative polymer precursors applied for olefin/paraffin separation. The various precursors and manufacturing processes are shown in Table 1.

### 3.1. Polyimide

Polyimide is a highly aromatic polymer commonly used as a precursor of CMS membranes due to its high carbon yield, which originates from its high glass-transition temperature (T_g_) and rigid structure. In addition, polyimide has the advantage of being able to tune various chemical structures, which facilitates the change of properties accordingly. Kapton and Matrimid (commercial polyimides) have frequently been used for CMS membranes, and synthesized polyimide precursors based on 6FDA and BPDA have been reported.

#### 3.1.1. Matrimid

**Figure 6:**
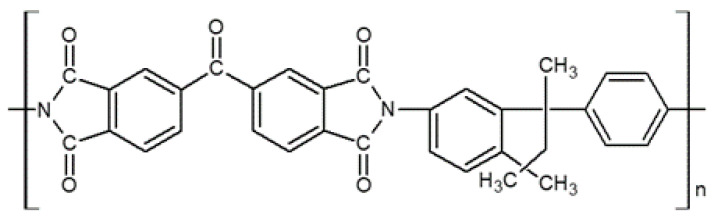
Chemical structure of the Matrimid polyimide precursor.

Matrimid is a commercial polyimide that has been widely used in the field of gas separation due to its high thermal stability and processability (Figure 6). In particular, it is a very attractive material for polymeric membranes and provides excellent permeability and selectivity. These advantages allow the CMS membrane to offer excellent performance. Thus, this representative, useful polymer precursor has been studied by many researchers.

Steel et al. prepared CMS membranes derived from Matrimid precursors pyrolyzed at different temperatures and characterized their micropore structures. The high pyrolysis temperature of 800 °C significantly reduced the ultramicropore size, which excludes the passage of both C_3_H_6_ and C_3_H_8_, resulting in poor separation properties. The lower final temperature of 500 °C led to more attractive transport due to its more open structure, resulting in C_3_H_6_ permeance of 13 barrer and selectivity of 40. Furthermore, the slit-like carbon structure was suitable for C_3_H_6_ and C_3_H_8_ molecules of a compact nature [89]. In addition, the C_2_H_4_ permeance of a Matrimid-based CMS membrane could be increased by accelerating the heating rate without the loss of selectivity. The fast pyrolysis induced a shorter densification period of the carbon matrix and a larger mass loss compared to the existing pyrolysis protocol, resulting in slightly higher permeability. The CMS membrane showed high entropic selectivity that enabled diffusion (favoring the slimmer C_2_H_4_ molecule) across the carbon layer of rigid slit-like structures [60].

**Figure 7:**
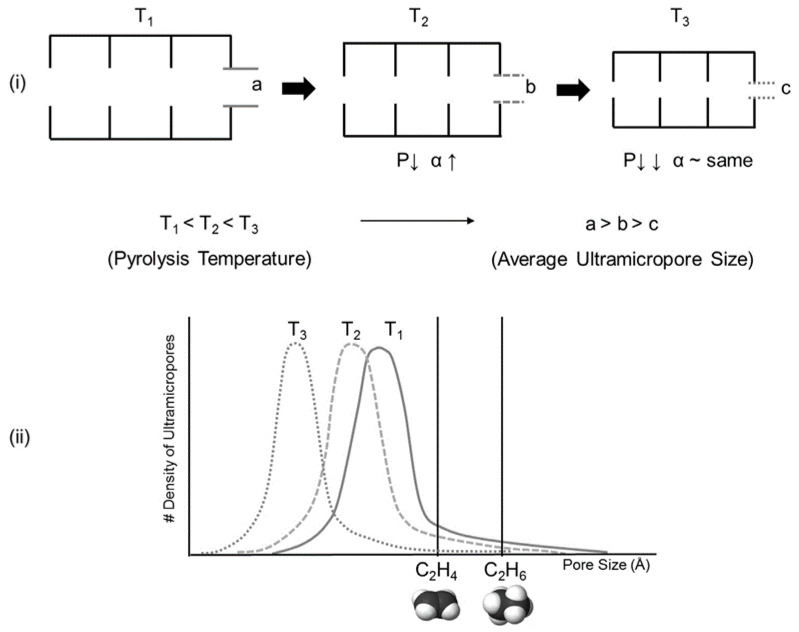
Effect of pyrolysis temperature on (**i**) CMS structure and (**ii**) Ultramicropore distribution. (α = flow direction). Adapted from [81] with permission. Copyright (2015) Elsevier.

The pore structure of a Matrimid-based CMS membrane was studied using a method based on differently sized gas probes as well as XRD, positron annihilation lifetime spectroscopy (PALS), and CO_2_ sorption. At a high pyrolysis temperature, the d-spacing of the CMS membrane (~3.8 Å) became narrower and a large number of smaller pores (<4.2 Å) were developed. Furthermore, the corrected diffusivity trends showed that a majority of the ultramicropores in the CMS membrane were in the range 2.6–3.5 Å. In particular, the more sorptive and planar C_2_H_4_ can more easily penetrate the CMS membrane compared to round or bulky molecules such as CH_4_ and C_2_H_6_ (Figure 7) [81].

In another case, the separation performance of the CMS membrane derived from Matrimid dense film and asymmetric hollow fiber, was compared for C_2_H_4_/C_2_H_6_ separation. The selectivities of both were very similar (~12), but the thickness of the carbon active layer was very different. This difference was attributed to collapse of the porous, hollow-fiber substructure due to the relatively low glass-transition temperature (T_g_) of Matrimid. Nevertheless, this collapse phenomenon enabled the defective hollow fibers to transform into a highly selective CMS membrane during pyrolysis [62].

Commercial Matrimid material is expensive and the resulting CMS membrane is brittle and fragile. To overcome these problems, a CMS composite membrane on low-cost alumina hollow fiber was prepared. This provides high packing density, good mechanical strength, and lower material cost. The thin and defect-free CMS hollow fiber composite membrane derived from Matrimid was successfully prepared using a one-coating process. It showed good separation performance: 69.2 GPU and 18 for C_3_H_6_ permeance and C_3_H_6_/C_3_H_8_ selectivity, respectively [92].

#### 3.1.2. 6FDA-Based Polyimides

**Figure 8:**
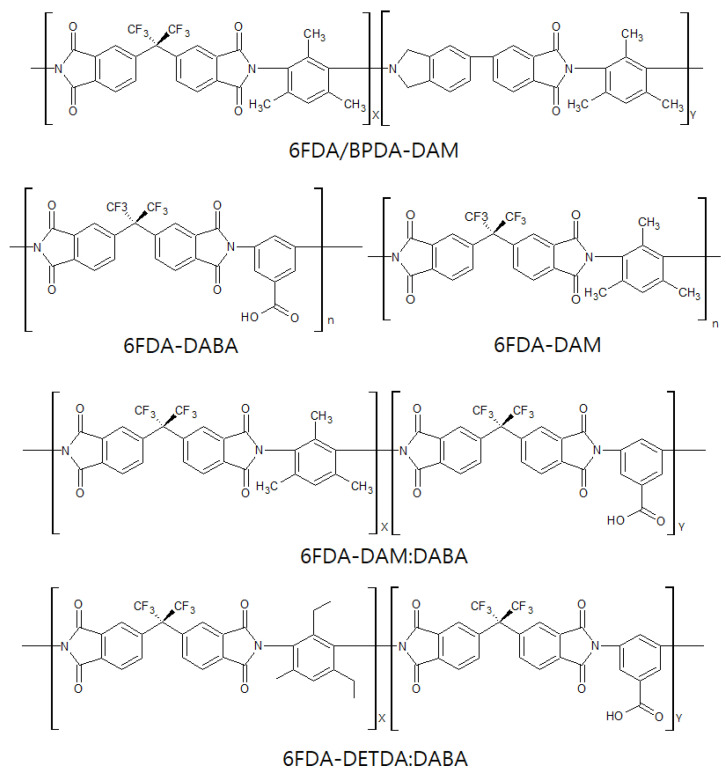
Chemical structure of 6FDA-based polyimide precursors.

The 6FDA-based polyimides are the polymer precursors most frequently used for olefin/paraffin separation. Studies on 6FDA-based CMS membranes have steadily progressed and are still reported to provide excellent performance for olefin/paraffin separation. The bulky –C(CF_3_)_2_- linkage of 6FDA-based polymers inhibits chain packing and offers a high fractional free volume, leading to high gas permeability [115]. In addition, it is believed that the bulky 6F group gives rise to a CMS membrane with higher gas permeability if derived from 6FDA-based polymers than if derived from Matrimid [116]. Furthermore, the 6FDA-based polyimides facilitate tuning of the chemical structure, which allows the polymer precursor to provide a variety of physical properties, as shown in Figure 8.

Steel et al. compared the carbon pore structures of CMS membranes derived from 6FDA/BPDA-DAM and Matrimid, and their performance for C_3_H_6_/C_3_H_8_ separation. The 6FDA/BPDA-DAM polyimide has an intrinsically less densely packed nature compared to Matrimid. This induces a greater cumulative pore volume (4–11 Å), resulting in higher permeability and lower selectivity. The CMS membrane derived from 6FDA/BPDA-DAM polyimide, after heating to 550 °C, showed very attractive performance of 200 barrer and 100 for C_3_H_6_ permeability and C_3_H_6_/C_3_H_8_ selectivity, respectively [88]. These polyimides were also compared using a gas probe method to determine the transport properties of the CMS membranes and differences in their ultramicropore distributions. These researchers mentioned that the structure of the polymer precursor material has a non-trivial connection with the resulting performance [85]. Furthermore, Xu et al. reported that a CMS membrane derived from 6FDA/BPDA-DAM hollow fiber maintained a better asymmetric structure during pyrolysis, while a substructure collapse with related loss of permeance was found in a Matrimid-based CMS membrane. This is attributed to a higher T_g_ (424 °C) and greater rigidity of the 6FDA/BPDA-DAM polyimide, leading to a thin active carbon layer and higher permeance [90].

**Figure 9:**
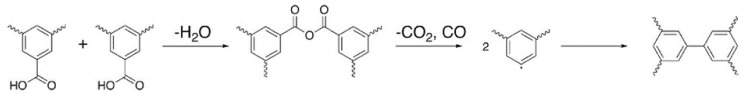
Thermal crosslinking mechanism of 6FDA-DABA. Adapted from [93] with permission. Copyright (2019) Elsevier.

Pre-crosslinking of a DABA-containing 6FDA-based polyimide at a temperature below T_g_ has been done [117,118]. This improved the chemical and physical stability of the polymer material and resulted in an increase of the gas permeability of the polymeric membrane. The crosslinkable 4,4′-(hexafluoroisopropylidene) diphthalic anhydride-3,5-diaminobenzoic acid (6FDA-DABA) polyimide was utilized to prepare CMS membranes (Figure 9) [93]. The pre-crosslinked CMS membrane possessed higher graphitic carbon content, which induced π-π interaction between the C_3_H_6_ and graphitic carbons, resulting in remarkably increased C_3_H_6_/C_3_H_8_ selectivity.

**Figure 10:**
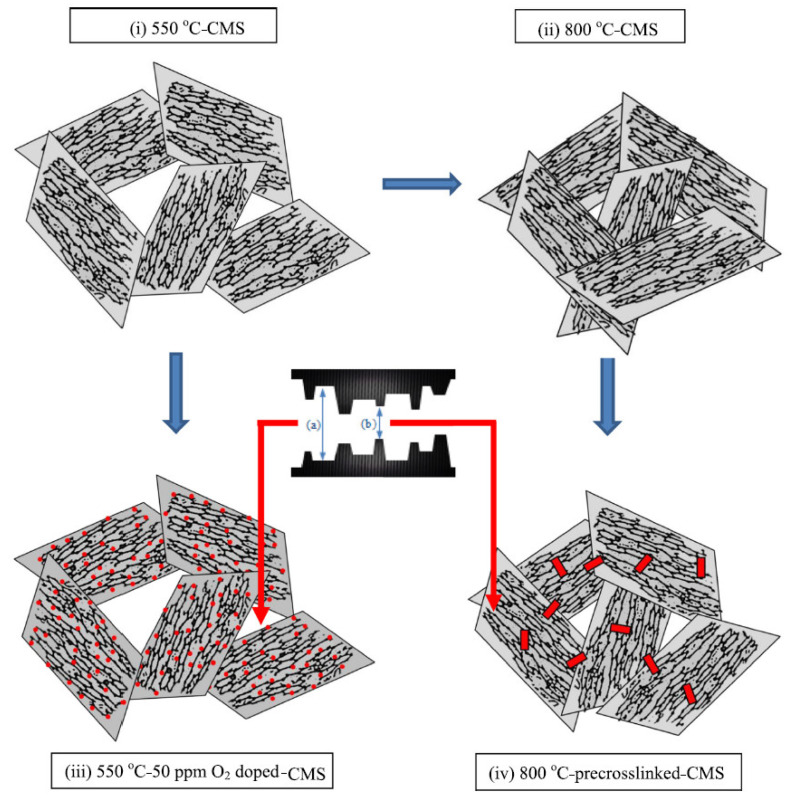
Effects of pyrolysis temperature, O_2_ doping, and pre-crosslinking on CMS structures; (**a**) micropores and (**b**) ultramicropores. Adapted from [77] with permission. Copyright (2016) Elsevier.

Fu et al. investigated the effect of O_2_ doping and pre-crosslinking of 4,4′-(hexafluoroisopropylidene) diphthalic anhydride/2,5-diethyl-6-methyl-1,3-diaminobenzene-3,5-diaminobenzoic acid (6FDA/DETDA-DABA)-based CMS membranes on their performance for C_3_H_6_/C_3_H_8_ separation [77]. The use of O_2_ doping as a fine-tuning method gave rise to a narrower ultramicropore structure in the CMS membrane and enhanced C_3_H_6_/C_3_H_8_ selectivity by more than 50. With pre-crosslinking, limiting the movement of polymer chains creates microvoids and packing disruptions, as shown in Figure 10.

This structure might be maintained after the subsequent pyrolysis; therefore, the available sorption sites could be increased and the permeability significantly enhanced with only a slight loss of selectivity.

CMS membranes derived from 6FDA-based polyimide have also been developed on inorganic supports due to its brittleness and handling issues in practical applications of non-supported CMS membranes. The inorganic support can provide mechanical strength to a CMS membrane. Furthermore, the collapse of the porous substructure of asymmetric CMS hollow fibers was often reported, which collapse causes reduction of gas permeance. Ma et al. prepared 6FDA-based CMS composite membranes on an alumina disc with intermediate layer [42]. The CMS membrane showed a C_3_H_6_ permeance of 9 GPU and C_3_H_6_/C_3_H_8_ selectivity of 36. Moreover, the effects of feed pressure, gas fraction, operating temperature, and carbon layer thickness on the C_3_H_6_/C_3_H_8_ separation performance were thoroughly studied [75,96].

Therefore, a CMS membrane that exceeds the upper bound performance and has excellent stability is promising either to replace or to support, the energy-intensive distillation process. However, it is difficult to completely replace the conventional distillation process with membrane-only processes due to limitations on separation performance. Some researchers reported using an advanced hybrid membrane-distillation process and 6FDA-based CMS membrane with enhanced olefin/paraffin separation performance. L. Xu et al. suggested a new hybrid process with an olefins-selective membrane unit and two distillation columns, or a series of olefin-selective membrane units and a distillation column [90]. The hybrid system can achieve significant energy savings and reduction of footprint in the hydrocarbon processes. Furthermore, a techno-economic analysis of the hybrid process (membrane followed by distillation) for C_3_H_6_/C_3_H_8_ separation resulted in a lower total cost of 13.1% compared to the conventional distillation process [94].

#### 3.1.3. Other Polyimides

The development of polyimide precursors based on BPDA was carried out for olefin/paraffin separation until the year 2000. BPDA could easily be imidized with diamines at high temperature, and the derived CMS membrane showed excellent separation performance after an additional oxidation process. Optimization of CMS membrane derived from 3,3′-4,4′-biphenyl tetracarboxylic dianhydride-4,4′-oxydianiline/diaminotoluene (BPDA-ODA/DAT) could be achieved by oxidation in air at 400 °C while the gas permeance of a CMS membrane without oxidation post-treatment was much lower [98]. J.I. Hayashi et al. studied the performance of CMS membranes derived from 3,3′-4,4′-biphenyl tetracarboxylic dianhydride-4,4′-oxydianiline (BPDA-ODA) for C_3_H_6_/C_3_H_8_ and C_2_H_4_/C_2_H_6_ separations according to the change of operating temperature. The selectivities for both increased from 4–5 and 25–29 to 5–7 and 33–56 for C_2_H_4_/C_2_H_6_ and C_3_H_6_/C_3_H_8_, respectively, while the gas permeance decreased [101]. The change of pore structure of a CMS membrane derived from BPDA-ODA according to pyrolysis temperature was also studied. The slit-like pores were gradually decreased with increasing pyrolysis temperature, resulting in the reduction of gas permeance. In addition, the post-oxidation process enhanced the gas permeance without the reduction of selectivity, which is attributed to increase of the micropore volume [99]. An asymmetric CMS hollow fiber membrane was prepared with polyimide precursor of 3,3′-4,4′-biphenyl tetracarboxylic dianhydride-dimethyl-3,7-diaminodiphenyl-thiophene-5,5-dioxide/3,5-diaminobenzoic acid (BPDA-DDBT/DABA). The asymmetric structure was maintained after carbonization, even though fusion of nodules was observed. The CMS membrane showed good stability and C_3_H_6_/C_3_H_8_ selectivity of 13 with high C_3_H_6_ permeance of 50 GPU [59].

**Figure 11:**
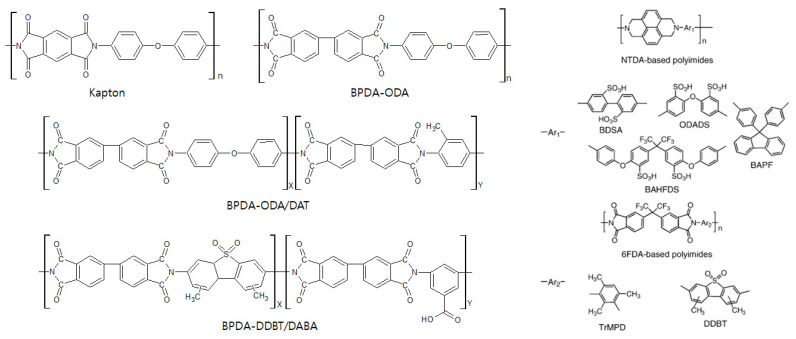
Kapton, and BPDA- and NTDA-based polyimide precursors. Adapted from [39] with permission. Copyright (2005) Elsevier.

In another study, CMS membranes derived from 1,4,5,8-naphthalene tetracarboxylic dianhydride (NTDA)-based sulfonated polyimides were prepared. The NTDA-based polyimide was synthesized by condensation polymerization and thermal imidization using chemicals with sulfonic groups such as benzidine-2,2′-disulfonic acid (BDSA), 4,4′-diaminodiphenylether-3,3′-disulfonic acid (ODADS), 9,9′-bis(4-aminophenyl)fluorene (BAPF), and 2,2-bis [4-(4-aminopheoxy)phenyl]hexafluoropropane disulfonic acid (BAHFDS). This resulted in the formation of NTDA-BAHFDS, NTDA-BAHFDS/BAPF, NTDA-BDSA/BAPF, NTDA-BDSA, and BTDA-ODADS polyimides, as shown in Figure 11. The large amount of microvoids caused by the template-like effect of sulfonic groups gave rise to higher gas permeability. Therefore, higher content of sulfonic groups led to increase in the gas permeability [39].

Kapton is another representative commercial polyimide precursor of CMS membranes, besides Matrimid. A CMS membrane made from Kapton polyimide precursor could be prepared without modification because it has homogeneous fine pores without defects such as cracks or large pores [119]. Suda et al. carried out mild activation of a Kapton-based CMS membrane at 400 °C under Ar gas to enlarge the pore size, and obtained the increased pore size distribution of 3.7–3.9 Å The modified CMS membrane showed high C_2_H_4_ and C_3_H_6_ permeance of 55.58 and 11.85 barrer and C_2_H_4_/C_2_H_6_ and C_3_H_6_/C_3_H_8_ selectivity of 5.82 and 25.27, respectively [97].

Recently, CMS membrane derived from hydroxyl polyimide (HPI-HD5) as a representative TR-able polyimide was introduced, but this is not for olefin/paraffin separation. The HPI-HD5 was thermally treated for thermally rearranged (TR) conversion and carbonized in IR furnace. The carbonization of polymer precursor in IR furnace containing oxygen gave rise to less processing time and energy requirement. This can improve the productivity by hundreds of times compared to that of conventional electrical furnace [120].

### 3.2. Phenolic Resin

Phenolic resin is a very attractive material for CMS membranes due to its thermosetting, high heat resistance, and carbon yielding properties [121]. Furthermore, it is a very inexpensive polymer. For these reasons, it is applied in a variety fields. This attractive polymer is being used as a precursor to create CMS membranes for gas separation, but in some studies, these membranes have been used for olefin/paraffin separation.

A thermal curing process is typically part of the preparation of CMS membranes derived from phenolic resin; this provides high thermal resistance and maintains the integrity of the membrane during pyrolysis. Fuertes et al. prepared CMS membranes derived from phenolic resin cured in air at 150 °C for 2 h on alumina tubular supports [74,102,103,106,107]. In addition, pre-oxidation and post-oxidation were carried out until reaching 400 °C. The former creates the O_2_ bridge between aromatic molecules, which results in more open porosity and in better performance for olefin/paraffin separation. The latter enhances the surface diffusion of condensable hydrocarbon by adsorption in micropores instead of via the molecular sieve mechanism [74,102,107]. These researchers also systematically studied the effects of pyrolysis temperature, heating rate, soaking time, and atmosphere on the separation performance of CMS membranes [106].

### 3.3. Polymer of Intrinsic Microporosity (PIM)

**Figure 12:**
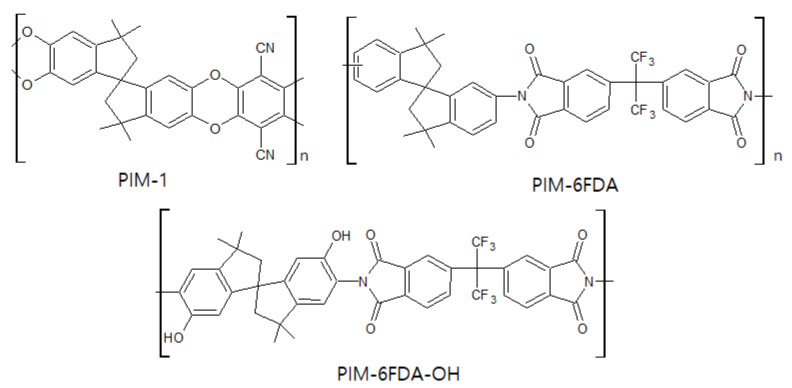
Chemical structures of PIM-based polymer precursors.

PIM is a relatively new, state of the art material for CMS membranes, as well as a polymer membrane used for gas separation. As shown in Figure 12, this polymer has a rigid backbone that induces inefficient packing of chains in the solid state, which condition provides high surface area, high free volume, and undetectable T_g_ [108,110]. These properties enable it to provide excellent separation performance with good flexibility and processability. A PIM-1-based CMS membrane was prepared by heating in the range 400–800 °C for C_2_H_4_/C_2_H_6_ separation. The C_2_H_4_ permeability was 44 and 1.3 barrer, and the C_2_H_4_/C_2_H_6_ selectivity was 6.29 and 13, at 600 and 800 °C, respectively [110]. To increase the separation performance of the PIM-based CMS membrane, additional study of the PIM precursor was carried out. A CMS membrane derived from PIM-6FDA-OH showed higher C_2_H_4_ permeability (10 barrer) and C_2_H_4_/C_2_H_6_ selectivity of 17.5 compared to that of the PIM-1-based CMS membrane [111]. Furthermore, the C_3_H_6_/C_3_H_8_ separation performances of thermally rearranged (TR) and CMS membranes derived from PIM-6FDA-OH were compared. The CMS membrane showed higher C_3_H_6_ permeance (45 barrer) and C_3_H_6_/C_3_H_8_ selectivity (33) than the TR membrane did [112]. After an OH-free PIM-6FDA was carbonized at 800 °C, it showed a more ordered carbon structure compared to a CMS membrane derived from PIM-6FDA-OH, which indicated increased graphitization. The more ordered graphitic carbon structure enhanced C_2_H_4_/C_2_H_6_ selectivity up to ~16 [109].

**Figure 13:**
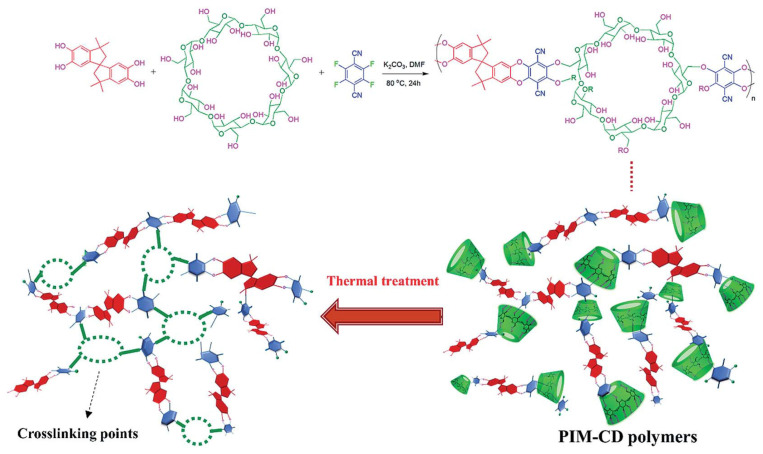
Synthesis and thermal crosslinking of a PIM-CD polymer precursor. Adapted from [122] with permission. Copyright (2017) Royal Society of Chemistry.

Liu et al. prepared PIM-cyclodextrin (CD) via nucleophilic substitution copolymerization and then thermally treated the polymer in the range 300–600 °C [122]. With increase in the thermal treatment temperature, the thermally liable CDs were decomposed. The CD cavities became micropores after thermal-treatment, which crosslinked points in the polymer matrix. The 3-D network provided selective, narrow gates without loss of gas diffusion (Figure 13). However, there was concern about severe packing and shrinkage of micropores and ultrafine micropores by excessive crosslinking and carbonization at high temperature because these could decrease the gas permeability. The CMS membrane derived from PIM-CD at 400 °C showed very high C_3_H_6_ permeability (2093 barrer) with low C_3_H_6_/C_3_H_8_ selectivity (5.19).

### 3.4. Other Polymers

**Figure 14:**
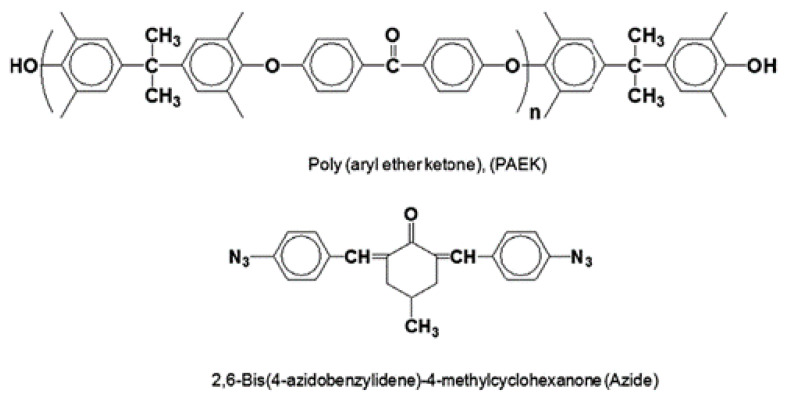
Chemical structures of PAEK and Azide. Adapted from [113] with permission. Copyright (2009) Elsevier.

Many other polymer precursors, such as polyacrylonitrile (PAN), polyfurfuryl alcohol (PFA), polyetherimide (PEI), poly(2,6-dimethyl-1,4-phenylene oxide) (PPO), and Poly(phthalazinone ether sulfone ketone) (PPESK), in addition to the aforementioned polymers, have also been used for gas separation. However, among them, only a few precursors were reported useful for olefin/paraffin separation due to their complex synthetic processes, relatively low free volume, poor separation performance, and poor processability. The polymer poly (aryl ether ketone) (PAEK) formed a semi-interpenetrating network (IPN) with 2,6-bis(4-azidobenzylidene)-4-methyl-cyclohexanone (Azide) as a photosensitive crosslinker, and PEAK-Azide was used as the precursor of a CMS membrane (Figure 14) [113]. The semi-IPN matrix provided stronger thermal stability and higher carbon yield than pure PAEK did. In addition, the CMS membrane derived from PAEK-Azide (80:20) showed good separation performance of 48 barrer and 44 for C_3_H_6_ permeability and C_3_H_6_/C_3_H_8_ selectivity, respectively. Moreover, this precursor is a less expensive material than polyimide is.

Richter et al. prepared a CMS membrane derived from a polyester-resin precursor prepared by crosslinking unsaturated polyester and styrene with a 3-D network [76]. The CMS membrane, which had a very thin carbon layer (125 nm) on an alumina tubular support, was applied for C_4_H_8_/C_4_H_10_ separation. Furthermore, the CMS membrane O_2_-treated at 350 °C showed very high C_4_H_8_ permeance (574 GPU). However, low C_4_H_8_/C_4_H_10_ selectivity (1.93) was observed because this membrane was prepared for H_2_/hydrocarbon separation.

### 3.5. Inorganic-Containing Polymers

To improve their separation performance and physical properties, inorganic nanoparticles, MOFs, and carbon-based nanomaterials have been introduced into the matrix of membranes. Incorporating such inorganic materials into CMS membranes has been also employed. Teixeira et al. reported a CMS membrane derived from phenolic resin precursor loaded with boehmite nanoparticles. The boehmite was transformed to alumina via dihydroxylation at high temperature, which process provided a high carbon yield. This membrane exhibited C_3_H_6_ permeance of 154.1 barrer and C_3_H_6_/C_3_H_8_ selectivity of 14.6, which surpassed the upper bound trade-off [105]. Furthermore, this research team varied the content of boehmite nanoparticles in the range 0.5–1.2 wt%. The higher carbon/alumina ratio increased the volume and average length of the micropores. The separation performance of a CMS membrane with the carbon/alumina ratio of 4.4 was 420 barrer and 18.1 for C_3_H_6_ permeability and C_3_H_6_/C_3_H_8_ selectivity, respectively [104].

**Figure 15:**
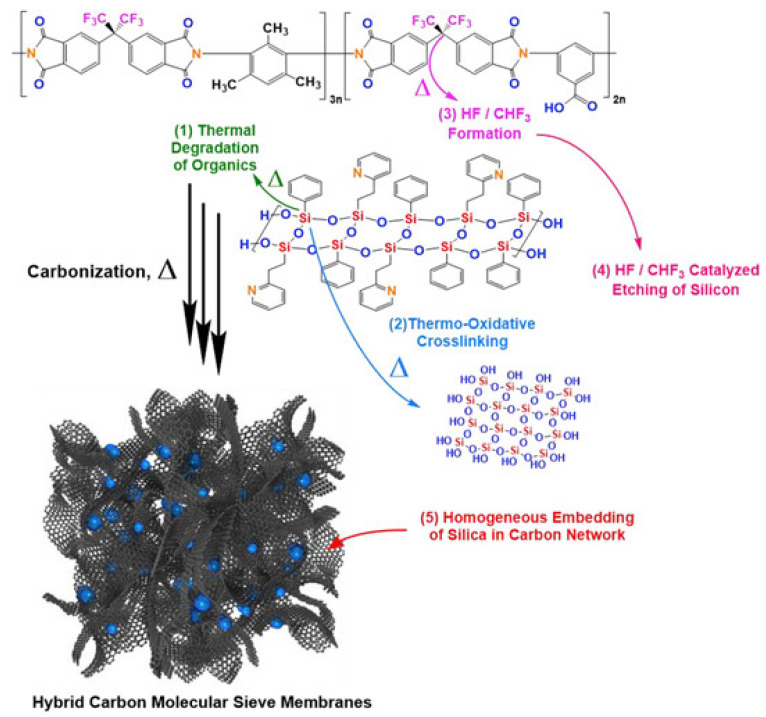
Nanoporous carbon-silica membrane derived from 6FDA-DAM/DABA and LPSQ. Adapted from [94] with permission. Copyright (2020) Elsevier.

For a carbon-silica membrane, a polyimide-silica precursor was prepared by mixing tetraethyl orthosilicate (TEOS, the silica precursor) and diethoxydimethylsilane (DEDMS, the silica-network modifier) in poly(acrylic acid) (PAA) synthesized from pyromellitic dianhydride (PMDA) and ODA. The well-dispersed spherical silica particles and the spaces between the carbon matrix and silica particles facilitated gas permeation. At the same time, a carbon matrix offered a selective domain, which provided good separation performance compared with other carbon membranes. The resultant gas permeability and selectivity were 398 barrer and 5.3 for C_2_H_4_/C_2_H_6_, and 375 barrer and 25.0 for C_3_H_6_/C_3_H_8_ separation [114]. A carbon-silica membrane was also fabricated with 6FDA-DAM/DABA polyimide and ladder-structured polysilsesquioxane (LPSQ) for C_3_H_6_/C_3_H_8_ separation, as shown in Figure 15. In this case, the densified and impermeable nonporous inorganic silica phase decreased C_3_H_6_ permeance, while C_3_H_6_/C_3_H_8_ selectivity significantly increased. This was attributed to its 6-fold enhanced diffusivity selectivity [94].

Various boron compounds with different molecular sizes were incorporated into a hydrolyzed PIM-1 as the polymer precursor [108]. The pore size of the boron-embedded CMS membranes increased, and the number of pores increased, due to the large size of the boron compound. This CMS membrane exhibited higher gas diffusivity and greater diffusion selectivity: C_2_H_4_ permeability of 13.7 barrer and C_2_H_4_/C_2_H_6_ selectivity of 9.7 in mixed gases.

**Figure 16:**
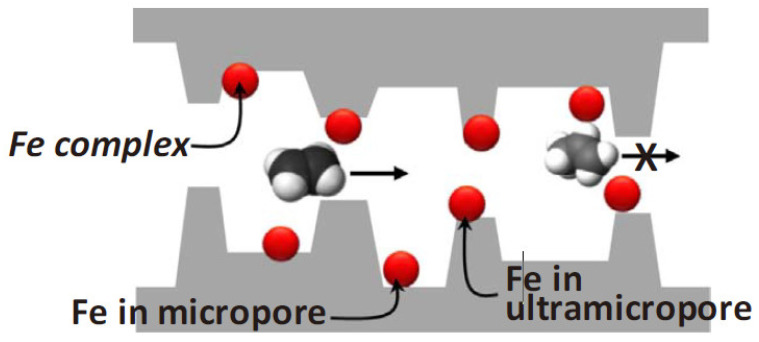
Fe complex in ultramicropores and micropores of Fe-containing CMS membranes. Adopted from [95] with permission. Copyright (2018) Elsevier.

In another case, Chu et al. prepared Fe-containing CMS membranes for olefin/paraffin separation [95]. They employed 1.1–3.2 wt% transition metal ions incorporated into the 6FDA-DAM/DABA polymer precursor and pyrolyzed under various conditions. The most suitable CMS membrane was obtained at the pyrolysis temperature of 550 °C using a rapid ramp rate due to the interaction between olefins and active Fe^2+^ cations. The slow-pyrolysis protocol can induce oxidation of Fe, which reduces the sorption selectivity. Furthermore, the Fe complex provided greater diffusion selectivity by blocking less selective ultramicropores, as shown in Figure 16, resulting in C_2_H_4_ permeability of 100 barrer and C_2_H_4_/C_2_H_6_ selectivity of 8.53. Performance of CMS membranes for olefin/paraffin separation was shown in Table 2.

## 4. Pyrolysis Process

Pyrolysis is the key parameter and a necessary process for CMS membranes. It produces the microporous carbon structure needed to provide the molecular sieving property. A variety of polymer precursors have been transformed through pyrolysis processes under different conditions. During pyrolysis, volatile chemical byproducts are typically emitted (i.e., NH_4_, CH_4_, H_2_, N_2_, CO, and CO_2_). The graphite-like structure derived from H_2_ evolution leads to significant weight loss of the polymer precursor. The heteroatoms in the polymer precursor are eliminated, which rearranges the molecular structure. Then, an amorphous, rigid, carbon matrix with a non-homogeneous, microporous structure is formed. The pore structure is generally determined by the polymer precursor and pyrolysis chemistry. Therefore, many researchers have been varying pyrolysis conditions such as temperature, heat ramping rate, thermal soaking time, and atmosphere as effective parameters [70]. To achieve excellent separation performance with a CMS membrane, these pyrolysis parameters should be optimized.

The pyrolysis temperature is the most effective parameter among the various pyrolysis conditions for predicting the final structure and separation performance of a CMS membrane. Pyrolysis is typically carried out in the range 500–1000 °C, which is in the range of decomposition and graphitization temperatures for the polymer precursors. A higher pyrolysis temperature leads to lower gas permeability and higher selectivity. This is attributed to greater compactness and crystallinity, which cause narrower interplanar spacing between the graphite-like layers in the carbon structure [123,124].

The heat ramping rate is generally maintained in the range 0.5–13 °C/min, which choice is related to the pore structure of the CMS membrane. The ramping rate determines the rate of progression of the polymer precursor into volatile components. As the heat ramping rate decreases, smaller pores and higher carbon crystallinity are obtained, while cracks, pinholes, distortions, and blisters may occur at faster heat ramping rates, lowering selectivity [125,126].

The soaking time is the amount of time a temperature is maintained after reaching the pyrolysis temperature. This parameter finely influences the transport property of a CMS membrane even though it is hard to expect big enhancement or change in terms of separation performance. Nevertheless, several researchers have studied variation of the heat ramping rate with long thermal soak times [106].

Pyrolysis is carried out in vacuum or under O_2_-free gases such as He, N_2_, and Ar to obtain a carbon structure without undesirable damage to the polymer precursor. The inert gases cause a CMS membrane to have a more open pore structure than occurs in vacuum. This is due to the higher gas-phase heat and mass transfer [127].

## 5. Aging and Stability Issues of CMS Membranes

### 5.1. Mechanical Stability

CMS membranes are undergoing a challenge of low mechanical strength due to brittle property after carbonization. This may be mitigated by optimizing the polymer precursor and preparation process [128,129,130]. However, enhancing the mechanical strength by these methods is limited because it is related to the performance of the CMS membrane.

The membrane configuration has significant impact on the mechanical stability of CMS membrane. The hollow fiber morphology which has relatively higher mechanical strength compared with flat sheet facilitates the modulation of CMS membranes, achieving the defect-free carbon layer as well as offering the high packing density [131]. In particular, supported CMS membrane is ideal to provide commercially-viable mechanical strength, which is favorable for operating at high temperature and high pressure. The substrate generally plays a role of mechanical support while the thin surface layer determines the permeability and selectivity of the membrane. The membrane thickness is main parameter to determine the transport rate. Therefore, composite CMS membrane can provide both high performances and mechanical stability.

On the other hand, a pre-crosslinking of polymer precursor can increase the flexibility of the CMS membrane. A crosslinked polymer generally becomes more brittle due to increase in the rigidity of the polymer chains. However, the CMS membrane undergoes the procedures of decomposition, aromatization, and fragmentation during pyrolysis, leading to rigid graphene-like layers with high fragility and brittleness [132]. This characteristic might be alleviated by the crosslinking between polymer chains, resulting in a more flexible CMS membrane. This crosslinking effect on CMS membrane was reported by Koh et al. for pre-crosslinked PVDF precursors [133].

### 5.2. Chemical Aging

**Figure 17:**
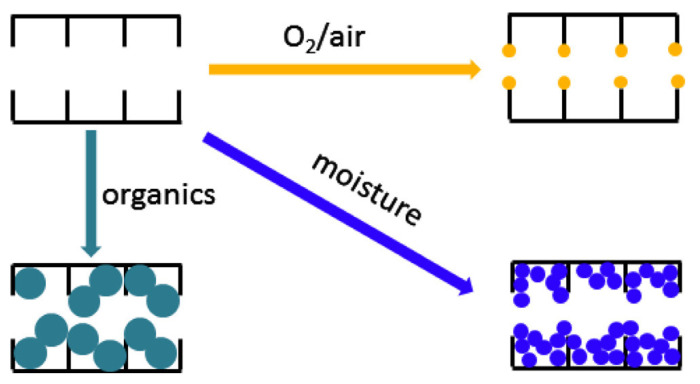
Mechanism of chemical aging in CMS membranes. Adapted from [91] with permission. Copyright (2014) Elsevier.

Chemical aging in a CMS membrane is induced by adsorption or interaction with external species such as organic contaminants, O_2_ (or air), and humidity, as illustrated in Figure 17. This causes severe degradation of gas permeation due to plugging of the pore structure [91].

For chemical aging by organic contaminants, Jones et al. exposed CMS membranes to hexane, vacuum pump oil, phenol, and toluene, resulting in reduction of gas permeance due to the hydrophobic property of carbon [134]. However, the chemically aged pore structure of a CMS membrane could be regenerated simply by exposure to C_3_H_6_.

Menendez et al. investigated chemical aging by exposure to air, N_2_, and C_3_H_6_ of a phenolic resin-based CMS membrane. In particular, when the CMS membrane was exposed to O_2_, the permeance was significantly decreased, which may be attributed to reactive edge sites in the carbon structure for O_2_ chemisorption [103]. Moreover, the water was strongly adsorbed to the CMS membrane in high humidity, leading to the formation of water clusters on hydrophobic carbon pores. The adsorption of water significantly decreased gas permeance by reducing the available pore volume and pore size, as with aging by organic components.

Despite many attempted solutions aimed at recovering from the chemical aging of CMS membranes, this unwanted phenomenon is still a critical issue in their commercialization. A fundamental solution to chemical aging must be developed in further work.

### 5.3. Physical Aging

**Figure 18:**
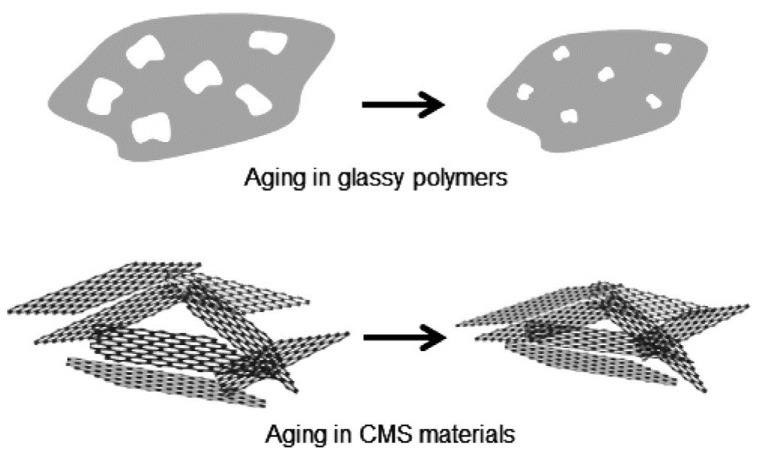
Physical aging of glassy polymers and CMS materials. Adapted from [91] with permission. Copyright (2014) Elsevier.

Recently, an unexpected form of permeance loss was observed while CMS membranes were stored under vacuum or in O_2_-free dry conditions. This observation was attributed to physical aging in 2014 by Xu et al. [91], as a possible explanation. Moreover, physical aging was observed in continuous gas permeation processes under O_2_-free conditions. This team hypothesized that CMS membranes undergo physical rearrangements to reach thermodynamic equilibrium due to initial imperfections of the graphene-like layers, as indicated in Figure 18. Physical aging was mainly caused during the early stage after pyrolysis. A similar phenomenon was observed by Lin et al. [75] in 2015. Raman spectroscopy of CMS membranes clearly revealed that the D/G ratio decreased after physical aging under pure N_2_ gas for one week. Such data indicates that the graphene-like layer of CMS membranes tends to rearrange into a more ordered structure.

Some efforts aimed at avoiding permeance loss by CMS membranes due to physical aging have been reported. CMS membranes derived from 6FDA-DABA could be thermally crosslinked at 350 and 450 °C prior to pyrolysis process. The pre-crosslinking of polymer chains increased T_g_ and restricted movements, leading to higher graphitic carbon content. This could prevent collapse of the pore structure in a CMS membrane, resulting in only 2% reduction of C_3_H_6_ permeability [93]. There are other studies that, even though they were not about olefin/paraffin separation, did involve efforts to prevent physical aging. Post-crosslinking of the CMS backbone was proposed to impede segmental movements (i.e., rearrangements and densification) of the graphene-like layers. This technique prevented physical aging to some extent, under a continuous active feed of 50 psia. However, a significant performance loss was still observed after 4 months of storage in vacuum [135]. In another case, a polydimethylsiloxane (PDMS) coating on the surface of a CMS membrane was employed to delay physical aging, even though this could not completely prevent the physical aging. The researchers mentioned that the PDMS layer may act as a resistance layer. A PDMS-coated CMS membrane showed a 35% reduction ratio of CO_2_ permeance while the original CMS membrane had 60% [136].

To apply CMS membranes at industrial scale, it is crucial to overcome this aging phenomenon to guarantee long-term operation. Based on the literature on this topic, it is clear that the shrinkage or densification of graphene-like layers as steps toward achieving a state of thermodynamic equilibrium must be prevented. Therefore, ways to modify or apply pre-/post-treatment of CMS membranes will be key technologies.

## 6. Conclusions and Prospects

Olefin is a useful product that has historically been separated from paraffin using an energy-intensive cryogenic distillation process. To replace this with alternative energy-effective, sustainable technologies, attempts involving such as adsorption, facilitated transport, and membranes have been reported. In particular, CMS membranes have proven attractive for olefin/paraffin separation due to their rigid, slit-like pore structure, which effectively allows the penetration and passage of the rather planar olefin molecule while blocking those of paraffin. Therefore, we reviewed the polymer precursors used to make CMS membranes for olefin/paraffin separation.

**Figure 19:**
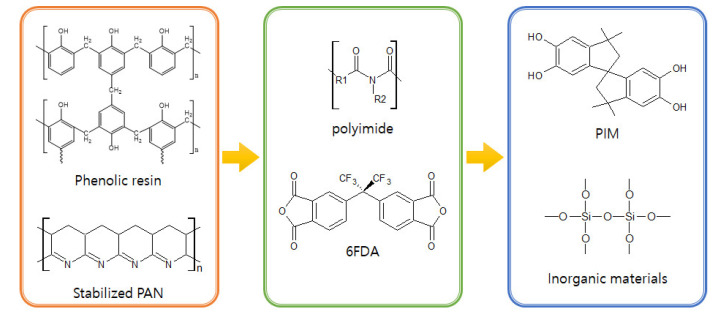
Progress of polymer precursor materials for CMS membrane.

**Figure 20:**
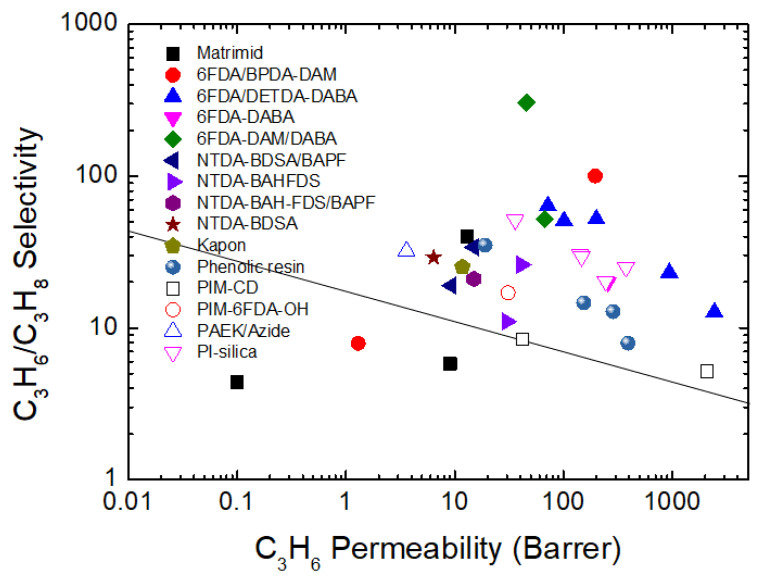
Upper-bound trade-off for CMS membranes derived from a variety of polymer precursors.

For olefin/paraffin separation, a variety of polymer precursors with a heating-resistant property have been employed. The progress of polymer precursor materials for CMS membrane was indicated in Figure 19. Initially, phenolic resin, PAN, and PPO with high aromatic carbon were typically used. In the 2000s polyimides such as Matrimid and 6FDA-based polymers have been commonly used as precursors of CMS membranes, which are polymers with relatively high thermal stability, good processability, and high free volume, which provide high carbon yield and gas permeability. In particular, 6FDA-based polyimide polymer with bulky -C(CF_3_)_2_-linkage is still the most widely used precursor with high separation performance as shown in Figure 20. PIM-based CMS membranes have recently been reported. These are favored due to their high free volume and undetectable T_g_ which are provided from rigid and twisted polymer chains. This give rise to excellent separation performance and good flexibility. For these reasons, polymers with high free volume and thermal stability are attracting much attention for CMS membranes. To provide even more beneficial properties to the polymers, several efforts have been carried out. These include such as chemical modification and addition of inorganic materials. These efforts enable better tuning of the ultramicropore and micropore carbon structures, and physical properties. This can enhance the separation performance and improves the stability of CMS membranes. However, the performance of CMS membrane for olefin/paraffin separation is relatively lower than with other inorganic membranes such as MOFs and organo-silica, even though CMS membranes have better processability. Therefore, polymer precursors with excellent physical properties should be investigated more, and the technologies needed for their fine-tuning must be further developed. The polymer precursor structure should be designed into higher.

The polymer precursor structure should be designed with a higher free volume and low structural deformation at high temperatures. The introduction of bulky CF_3_ group in 6FDA or twisted structure in PIM may be good examples. In addition, research on employing a filer that can provide higher porosity and physical properties while maintaining the pore structure of CMS membrane should be continuously studied.

On the other hand, the aging issues of CMS membranes have become a bottleneck to commercialization. In fact, chemical aging has been approached by many researchers over a long time, and a great deal of effort has been expended to solve the problem.

Nevertheless, a fundamental solution has not yet been proposed to completely stop aging. Furthermore, physical aging has recently become a major issue for CMS membranes. The degree of this unexpected phenomenon differs depending on the polymer precursors. Therefore, work must be carried out in the future to investigate physical aging of the various precursors and to propose a solution that stops or delays the loss of permeability.

## Figures and Tables

**Table 1:** Polymer precursors and processes for manufacturing CMS membranes.

Materials	Configuration	Pre-Treatment	Post-Treatment	Pyrolysis Temperature (°C)	Heating Rate (°C/min)	Soaking Time (h)	Atmosphere	Ref.
Matrimid	Film			550, 800	4	2, 8	Vacuum (<0.03 mmHg)	[88]
Matrimid	Film			550–800	0.25–13.3	2	Vacuum (<10 mtorr), Ar	[60]
Matrimid	Film			675	0.25–13.3	2	Ar	[85]
Matrimid	Film			550–800	4		Vacuum (0.03 mmHg)	[89]
Matrimid	Film, hollow fiber			550–800	0.25–13.3	2	Vacuum (<15 mtorr)	[62]
Matrimid	Hollow fiber			550	0.25–13.3	2	Ar (200 mL/min)	[90]
Matrimid	Hollow fiber			550	0.25–13.3	2	Ar (200 mL/min)	[91]
Matrimid	Alumina hollow fiber supported			650	2.8	1	He (100 mL/min)	[92]
Matrimid	Film			500–800	0.25–13.3	2	Vacuum, Ar	[81]
6FDA/BPDA-DAM	Film			550, 800	4	2, 8	Vacuum (<0.03 mmHg)	[88]
6FDA/BPDA-DAM	Film			675	0.25–13.3	2	Ar	[85]
6FDA-DETDA/DABA	Film		O_2_ doping	550–800	0.25–13.3	2	Ar	[77]
6FDA-DETDA/DABA	Film		Pre-crosslinking at 370 °C for 1.5 h	800	0.25–13.3	2	Ar	[77]
6FDA-DABA	Film	Crosslinking at 350–450 °C for 2 h		550	1	2	N_2_ (200 mL/min)	[93]
6FDA-DAM/DABA	Film, hollow fiber			675	0.25–10	2	Ar (400 mL/min)	[94]
6FDA-DAM/DABA	Alumina disc supported			550–750	4	2	Ar (100 mL/min)	[42]
6FDA-DAM	Film			550, 675	0.25–13.3	2	Ar (200 mL/min)	[95]
6FDA/BPDA-DAM	Hollow fiber			550	0.25–13.3	2	Ar (200 mL/min)	[90]
6FDA-DAM	Hollow fiber			550	0.25–13.3	2	Ar (200 mL/min)	[90]
6FDA/BPDA-DAM	Hollow fiber			550	0.25–13.3	2	Ar (200 mL/min)	[91]
6FDA-based polyimide	Hollow fiber			550	0.25–13.3	2	Ar (200 mL/min)	[91]
6FDA-based polyimide	Alumina disc supported	Pre-aging (oxidation)		550	4	2	Ar (100 mL/min)	[75]
6FDA-based polyimide	Alumina disc supported			550	4	2	Ar (100 mL/min)	[96]
Kapton	Film		Activation at 400 °C under Ar and He containing water vapor	1000	10	2	Vacuum (10^−5^ torr)	[97]
BPDA-ODA/DAT	Alumina tubular supported	Oxidation in air at 400–500 °C		500–700	5		N_2_ (100 mL/min)	[98]
BPDA-pp’ODA	Alumina tubular supported	Imidizing at 300 °C for 1 h	Oxidation using O_2_/N_2_ mixture or pure O_2_ for 3 h	600–900	5		N_2_	[99]
BPDA_aromatic diamine	Hollow fiber	Thermostabilization at 400 °C for 30 min in air		600–1000	15		N_2_ (2000 mL/min)	[100]
BPDA-based	Hollow fiber		Thermostabilization in air at 400 °C for 0.5 h	500–700	5		N_2_ (100 mL/min)	[59]
BPDA-pp’ODA	Film			370–450	5	1.5	N_2_ (100 mL/min)	[39]
NTDA-BDSA/BAPF	Film			700	5		Ar	[101]
Phenolic resin	Ceramic tubular supported	Curing at 150 °C for 2 h	Oxidizing with air at 300–400 °C for 30 min	700			Vacuum (<0.01 mbar)	[102]
Phenolic resin	Ceramic tubular supported	Curing at 150 °C for 2 h	Storage under air, N_2_, and C_3_H_6_	700			Vacuum (<0.01 mbar)	[103]
Phenolic resin	Alumina tubular supported			550	274	2	N_2_ (100 mL/min)	[104]
Phenolic resin	Alumina tubular supported			550	1	2	N_2_	[105]
Phenolic resin	Ceramic tubular supported			700–1000	0.5–10	1–8	Vacuum (<1 Pa), N_2_ (285 mL/min)	[106]
Phenolic resin	Alumina tubular supported	Air oxidative treatment at 250–400 °C	Air oxidative treatment at 300–400 °C	700			Vacuum (<0.01 mbar)	[107]
Phenolic resin	Alumina tubular supported	Curing at 150 °C for 2 h	Air oxidative treatment at 75–350 °C for 0.5 h	700			Vacuum (<0.01 mbar)	[74]
PIM-1	Film			500–900	1	1	Vacuum (<0.006 torr)	[108]
PIM-6FDA	Film			500–800	3	0.5	N_2_ (1000 mL/min)	[109]
PIM	Film	Annealing at 250 °C for 24 h		400–800	3	0.5	N_2_ (1000 mL/min)	[110]
PIM-CD	Film			300–600	1	2	Vacuum	[110]
PIM-6FDA-OH	Film			500–800	3	0.5	N_2_ (1000 mL/min)	[111]
PIM-6FDA-OH	Film			600	3	0.5	N_2_	[112]
PAEK/Azide	Film			450–650	0.2–2	2	vacuum	[113]
Polyimide	Film	Imidization at 100–300 °C for 1 h and 450 °C for 10 min		600	5	1	Inert gas (150 mL/min)	[114]
Polyester-resin	Alumina tubular supported		Oxidation at 300–400 °C for 0.5 h	700–800	1	0.5–1	Ar	[76]

**Table 2:** Performance of CMS membranes for olefin/paraffin separation.

Materials	Pyrolysis Temperature (°C)	Operating Temperature (°C)	Test Gas *	C_2_H_4_ Permeance **	C_2_H_4_/C_2_H_6_ Selectivity	C_3_H_6_ Permeance *	C_3_H_6_/C_3_H_8_ Selectivity	C_4_H_8_ Permeance *	C_4_H_8_/C_4_H_10_ Selectivity	Ref.
Matrimid	550	35	S			9.1 B	5.8			[88]
Matrimid	800	35	S			0.1 B	4.4			[88]
Matrimid	550	35	M	8.3 B	7.7					[60]
Matrimid	675	35	M	10.9 B	12.3					[60]
Matrimid	675	35	S	~18.0 B	~11.25					[85]
Matrimid	500	35	S			13 B	40			[89]
Matrimid	675	35	S	10 B	~12					[62]
Matrimid	700	35	S	0.25 G	~12					[62]
Matrimid	550	35	S	2.1 G	4	0.76 G	21			[90]
Matrimid	550	35	M	5.5 G	3.1					[91]
Matrimid	650	25	M			69.2 G	18			[92]
Matrimid	500	35	S	~78.9 B	~3.96					[81]
Matrimid	550	35	S	~18.7 B	~6.31					[81]
Matrimid	675	35	S	~17.7 B	~10.4					[81]
6FDA/BPDA-DAM	550	35	S			196 B	100			[88]
6FDA/BPDA-DAM	800	35	S			1.3 B	7.9			[88]
6FDA/BPDA-DAM	675	35	S	~58.7 B	~7.63					[85]
6FDA/DETDA-DABA	550	35	M			2444 B	12.7			[77]
6FDA/DETDA-DABA (O2 doping)	550	35	M			941 B	23.1			[77]
6FDA/DETDA-DABA (O2 doping)	550	35	M			101 B	50.7			[77]
6FDA/DETDA-DABA	800	35	M			71 B	63.7			[77]
6FDA/DETDA-DABA (pre-crosslinking)	800	35	M			200 B	52			[77]
6FDA-DABA	550	35	M			257 B	20			[93]
6FDA-DAM/DABA (embedding silica)	675	35	M			~67 B	~52			[94]
6FDA-DAM/DABA (embedding silica)	675	35	M			~4.1 G	~35			[94]
6FDA-based polyimide	550	25	M			9 G	36			[42]
6FDA-DAM/DABA (Fe loading)	550	35	M	10 B	11					[95]
6FDA-DAM/DABA (Fe loading)	550	35	M	~100 B	~8.53					[95]
6FDA-DAM/DABA (Fe loading)	550	35	S			~45.6 B	~304.9			[95]
6FDA/BPDA-DAM	550	35	S	15.9 G	3.9	17.5 G	3.9			[90]
6FDA-DAM	675	35	M	16.1 G	~4.8					[91]
6FDA/BPDA-DAM	550	35	M	8.8 G	3.9					[91]
6FDA-based polyimide	550	120	M			25.6 G	~13			[75]
6FDA-based polyimide	550	25	M			~9.94 G	~34			[75]
6FDA-based polyimide	550	RT	M			29.8 G	~31			[96]
BPDA-ODA/DAT	600	35	M			4.179 G	25			[98]
BPDA-ODA/DAT	600	100	M			18.5 G	18			[98]
BPDA-pp’ODA	700	100	S			~13.2 G	~19.1			[99]
BPDA-aromatic diamine	700	50	S	~8.69	~3.30					[100]
BPDA-aromatic diamine	850	80	S	~0.30 G	~7.26					[100]
BPDA-DDBT/DABA	600	100	M			51 G	12			[59]
BPDA-DDBT/DABA	600	100	M	110 G	3.1					[59]
NTDA-BDSA/BAPF	450	35	S	31 B	4.2	15 B	34			[39]
NTDA-BDSA/BAPF	450	35	M			9.3 B	19			[39]
NTDA-BAHFDS	450	35	S	66 B	3.5	41 B	26			[39]
NTDA-BAHFDS	450	35	M			30 B	11			[39]
NTDA-BAHFDS/BAPF	450	35	S	30 B	3.4	15 B	21			[39]
NTDA-BDSA	450	35	S	14 B	4.8	6.4 B	29			[39]
BPDA-pp’ODA	700	35	M			2.36 G	46			[101]
BPDA-pp’ODA	700	100	M			8.66 G	33			[101]
BPDA-pp’ODA	700		S	~40 B	~7					[101]
Kapton	400–1000	100	S	~55.5 B	~5.82	~11.8 B	~25.2			[97]
Phenolic resin	700	20 ± 1	S	~1.38 G	14					[102]
Phenolic resin	700	20 ± 1	S	~3027 G	1.3	~3092 G	1.45			[102]
Phenolic resin	700	20 ± 1	S	~49.85 G	2.35	~45.0 G	16.59			[103]
Phenolic resin	550	20	M			~286.6 B	~12.8			[104]
Phenolic resin	550	20	M			~392.6 B	~7.92			[104]
Phenolic resin (boehmite composite)	550	20	S			~154.1 B	14.6			[105]
Phenolic resin	800		M	16 B	5.4	19 B	35			[106]
Phenolic resin	700	20	S	~48.7 G	~3.75	~52.9 G	~33.1			[107]
Phenolic resin	700		S	8.65 G	2.2	84.4 G	11.4			[74]
PIM (boron-doped)	700	35	M	~13.6 B	~9.69					[108]
PIM-6FDA	800	35	M	3.02 B	17.9					[108]
PIM-1	600	35	S	44 B	6.29					[110]
PIM-1	800	35	S	1.3 B	13					[110]
PIM-CD	400	35	S			2093 B	5.19			[122]
PIM-CD	600	35	S			42 B	8.4			[122]
PIM-6FDA-OH	800	35	S	10 B	17.5					[111]
PIM-6FDA-OH	800	35	M	~10 B	14					[111]
PIM-6FDA-OH	600	35	M			31 B	17			[112]
PAEK/Azide	550	35	M			3.6 B	32			[113]
PI (silica dispersion)	600	25	S	40 B	13.3	36 B	51.4			[114]
PI (silica dispersion)	600	25	S	150 B	8.3	143 B	30.4			[114]
PI (silica dispersion)	600	25	S	280 B	5.4	244 B	20.3			[114]
PI (silica dispersion)	600	25	S	155 B	7.8	147 B	29.4			[114]
PI (silica dispersion)	600	25	S	398 B	5.3	375 B	25			[114]
Polyester-resin	800	150	S					~574.0 G	~1.93	[76]

* S and M indicate single and mixture, respectively. ** B and G indicate barrer and GPU, respectively.

## Data Availability

Not applicable.
